# Variation in breeding phenology in response to climate change in two passerine species

**DOI:** 10.1007/s00442-022-05306-5

**Published:** 2022-12-22

**Authors:** Fredrik Andreasson, Andreas Nord, Jan-Åke Nilsson

**Affiliations:** Department of Biology, Section for Evolutionary Ecology, Lund University, Ecology Building, 223 62 Lund, Sweden

**Keywords:** Breeding phenology, Breeding season length, Climate change, Laying date, Life history

## Abstract

Increasingly warmer springs have caused phenological shifts in both plants and animals. In birds, it is well established that mean laying date has advanced to match the earlier food peak. We know less about changes in the distribution of egg-laying dates within a population and the environmental variables that determine this variation. This could be an important component of how populations respond to climate change. We, therefore, used laying date and environmental data from 39 years (1983–2021) to determine how climate change affected laying date variation in blue tits (*Cyanistes caeruleus*) and marsh tits (*Poecile palustris*), two sympatric passerines with different life histories. Both species advanced mean laying date (0.19–0.24 days per year) and mean laying date showed a negative relationship with maximum spring temperature in both blue and marsh tits. In springs with no clear temperature increase during the critical time window (the time-window in which mean laying date was most sensitive to temperature) start of breeding in blue tits was distributed over a longer part of the season. However, there was no such pattern in marsh tits. Our findings suggest that temperature change, and not necessarily absolute temperature, can shape the variation in breeding phenology in a species-specific manner, possibly linked to variation in life-history strategies. This is an important consideration when predicting how climate change affects timing of breeding within a population.

**Supplementary Information:**

The online version contains supplementary material available at 10.1007/s00442-022-05306-5.

## Introduction

As spring temperatures increase due to climate change, plants and animals advance their reproductive phenology (Walther et al. 2002; Parmesan and Yohe 2003; Root et al. 2003). Plants respond to higher temperatures early in spring with earlier budding and flowering (e.g., Fitter and Fitter 2002). Primary consumers, like caterpillars, rely on this seasonal food source and need to synchronize their hatching with the phenology of their host plants. Secondary consumers, like caterpillar-eating birds, must, in turn, synchronize their breeding with the earlier peak in food abundance to meet offspring demand (e.g., Lack 1968; van Noordwijk et al. 1995). This match (or mismatch) between start of breeding and food abundance has often been studied using insectivorous birds in the northern temperate zone. These studies show that many species have advanced their egg laying (Crick et al. 1997; McCleery and Perrins 1998; Crick and Sparks 1999; Dunn and Winkler 1999; Sanz 2002; Visser et al. 2006; Charmantier et al. 2008; Goodenough et al. 2010; Källander et al. 2017; Bailey et al. 2022) to match changes in temperature (Schaper et al. 2012), in vegetation (Myneni et al. 1997) and, consequently, caterpillar phenology (Visser et al. 1998, 2006; Buse et al. 1999; van Asch et al. 2013; Burgess et al. 2018).

Most studies on climate change-effects on laying date have focused on changes of central tendencies, i.e., mean or median breeding start (Crick et al. 1997; McCleery and Perrins 1998; Crick and Sparks 1999; Dunn and Winkler 1999; Sanz 2002; Visser et al. 2006; Charmantier et al. 2008; Goodenough et al. 2010; Källander et al. 2017; Bailey et al. 2022). However, climate change could also shape the distribution of individual laying dates within or between populations. For example, a warming climate could result in a shortening of the breeding season if late-breeding individuals can advance laying more than early breeders. In a long-term study of 73 bird species in the boreal zone in Finland, Hällfors and colleagues (2020) found that 31% of species have shortened their breeding seasons over the last four decades. This could indicate that early-laying individuals are more constrained by environmental cues other than temperature compared to later-laying individuals. A shortening of the length of the breeding season could also be observed if higher spring temperatures reduce the duration of peak food availability (Buse et al. 1999; Smith et al. 2011; Burger et al. 2012), thus constraining the range of laying dates that maximize reproductive success. In line with this, studies on single-brooded species in the Northern Hemisphere show that laying dates vary less in years with high average spring temperatures (Winkler et al. 2002; Møller et al. 2010; Halupka and Halupka 2017). Multi-brooded species, on the other hand, often utilize a wider range of prey species and might be able to prolong their breeding season in a warming climate (Møller et al. 2010; Halupka and Halupka 2017).

Individuals that breed too late in relation to the food peak must feed their nestlings with a steadily declining supply of food (Siikamäki 1998), which can have negative effects on offspring condition (Burger et al. 2012; Samplonius et al. 2016). The consequences of mismatch for nestling fitness are species-specific and depend on both habitat selection and use of alternative prey (Veen et al. 2010; Burger et al. 2012; Samplonius et al. 2016) but, in general, recruitment in single-brooded species declines with laying date (Perrins 1965; Verhulst and Tinbergen 1991; Verboven and Visser 1998; Charmantier et al. 2008; Reed et al. 2013; Ramakers et al. 2020). However, selection on timing of breeding can differ between species where early breeding in relation to conspecifics is more important than synchrony (in terms of matching the food peak) and vice versa (Pakanen et al. 2016). Here, we analyzed if this theoretical framework can be applied to two sympatric passerines, blue tits (*Cyanistes caeruleus*) and marsh tits (*Poecile palustris*), that have different timing of breeding and potentially different fitness functions in relation to spring temperature. Despite being similar in many aspects, the two species differ markedly in social organization during the non-breeding season. Marsh tits aggregate in small flocks that defend a territory during the entire winter (Nilsson and Smith 1988) while blue tits are less territorial and instead forage over large home-ranges within mixed-species flocks (Perrins 1979). Thus, being able to fledge early is an important trait for marsh tits that need to secure their establishment in a winter flock (Nilsson and Smith 1988) but is presumably less advantageous for blue tits.

We used data collected over four decades to assess how spring temperature influences timing of breeding and how the temporal change in ambient temperature each spring affects the variation in laying date within a season in the two tit species. Schaper and colleagues (2012) showed that temperature increase, and not just temperature itself, during the time-period before egg-laying has a direct, positive effect on earlier laying in great tits (*Parus major*). Since the study by Schaper and colleagues was an indoor experimental study, the effect could be proved to act independently of phenological responses on other trophic levels (i.e., plant and caterpillar phenology). This highlights the need for including also rate of temperature increase in models of phenological advancement of egg-laying. On this basis, we predict that laying dates will vary more within the population in years when this direct cue is lacking (i.e., when temperature slope is close to zero) due to individual uncertainty in when to lay. The inclusion of both blue tits and marsh tits allowed us to evaluate this possibility within a life-history context.

## Methods

### Study population

We used 39 years of breeding data (1983–2021) from blue tits and marsh tits in a nest-box breeding population in southern Sweden, 20 km outside of Lund (55°42ʹN, 13°28ʹE, study site no. 5 in Källander et al. 2017). The population contains over 500 nest-boxes and annual mean population size (± SD) over the years was 138 ± 55 (range 37–243) and 40 ± 10 (range 23–63) breeding pairs for blue- and marsh tits, respectively. Laying date (the day when the first egg is laid) was determined by checking nest-boxes at least once weekly during egg-laying, and back-calculating assuming one egg was laid per day. To exclude all breeding events that where re-nestings or second broods, we removed all events where the first egg was laid more than 31 days after the first egg in the population as we considered such breeding attempts to be outside of the natural variation in timing of the first egg. In each year all documented nests with laid eggs were used to calculate mean laying date and variation in laying date (1 SD) for the population. Yearly mean laying date was rounded to the nearest integer. Unfortunately, we do not have individual ID for females in all years and all analyses were therefore based on mean yearly laying dates and variation in laying dates at the population level.

### Advancement of mean laying date over time

To provide a comparison to other studies, we began by assessing the change over time by analyzing mean laying date as a function of year in a linear model.

### Sliding window analysis

To identify the environmental cues and the time-window that explained most of the variation in mean laying date, we adopted a sliding-window approach, using the R package *climwin* (Bailey and van de Pol 2016; van de Pol et al. 2016). Daily mean, maximum and minimum air temperatures (°C), and daily precipitation (mm d^−1^), recorded by a weather station in Lund (Swedish Meteorological and Hydrological Institute, 2021, unpublished data) were used as environmental variables. For detailed information about the *climwin* analyses, see the electronic supplementary material. For models on mean laying date, model residuals were weighted by the inverse of the standard error of mean laying date to account for uncertainty in the estimation of this variable, which can be influenced by variation in sample size between years.

### Laying date distribution

Once the best time-window and climate signal for explaining variation in mean laying date was identified (Table S1), we used linear models to estimate how the candidate signal within the selected time-window related to variation in laying date (1 SD). As the change in signal across the time-window could affect laying date (e.g., Schaper et al. 2012), we also added the change in signal (i.e., slope) to our models. Firstly, we tested whether the response in laying date variation differed between species by running two models: one that included the three-way interaction between species, climate signal and slope and one that only contained the two-way interaction between either climate signal and species or signal slope and species. The simpler model provided a better fit (ΔAICc = 15.4) and was used to test if the two climate variables (signal and slope) differed between species (Table S2). We then proceeded to analyze each species separately where we regressed both linear and quadratic effects of the candidate signal and its slope on variation in laying dates (also including the interaction between climate signal and slope). There was no correlation between climate signal and slope for either species (Fig. S1).

Models were run using *lm* in base R (R Core Team, 2022). To be able to evaluate potential linear effects without removing the quadratic term, all models with climate variables were run using orthogonal polynomials.

## Results

### Advancement of mean laying date over time

**Fig. 1:**
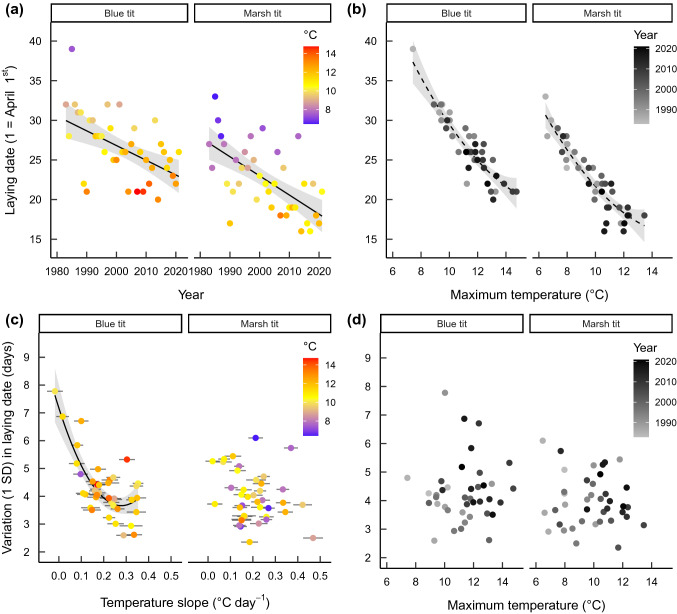
Mean laying date (1 = April 1st) as a function of **a** time and **b** maximum spring temperature in the selected time-window; and laying date variation (1 SD) as a function of **c** temperature change over the selected time-window, and **d** maximum spring temperature in the selected time-window, in blue tits and marsh tits. Regression lines are linear in **a** and quadratic in **b** and **c**. Dashed lines in **b** indicate *p* = 0.057 and *p* = 0.050 for the quadratic term in blue tits and marsh tits, respectively. Horizontal error bars in **c** represent ± SE for the estimated temperature slope. Shaded bands are 95% confidence intervals. Colors in **a** and **c** represents the maximum spring temperature within the selected time-window as indicated in the legend, and the greyscale in **b** and **d** represents year of study (1983 to 2021)

Mean laying date advanced 0.185 (± 0.049 SE) and 0.242 (± 0.046 SE) days per year in blue and marsh tits, respectively, over the study period. Thus, mean laying date have advanced 7.2 and 9.4 days since 1983 (*p* < 0.001, Fig. 1a, Table 1).

**Table 1:** Estimates, test statistics and significance values from models on mean laying date and variation in laying date (1 SD) in blue tits and marsh tits over the 39-year study period. Temperature variables were calculated within the selected time-window separately for each species. Significance values where *p* < 0.05 are given in bold font and where 0.1 > *p* > 0.05 in italics

Variable	Estimate (SE)	df	t	*p*
**Blue tit**				
*Advancement of mean laying date over time*				
Year	− 0.185 (0.049)	1, 37	− 3.8	**0.0005**
*Mean laying date vs maximum temperature*				
Temperature	− 22.5 (1.7)	1, 36	− 13.0	** < 0.0001**
Temperature^2^	3.5 (1.8)	1, 36	2.0	*0.057*
*Variation in laying date vs maximum temperature*				
Temperature	0.7 (0.7)	1, 34	1.1	0.30
Temperature^2^	0.7 (0.7)	1, 34	1.0	0.31
Temperature slope	− 4.4 (0.7)	1, 34	− 6.5	** < 0.0001**
Temperature slope^2^	3.2 (0.7)	1, 34	4.5	** < 0.0001**
**Marsh tit**				
*Advancement of mean laying date over time*				
Year	− 0.242 (0.046)	1, 37	− 5.3	** < 0.0001**
*Mean laying date vs maximum temperature*				
Temperature	− 23.2 (1.6)	1, 36	− 14.3	** < 0.0001**
Temperature^2^	3.3 (1.6)	1, 36	2.0	*0.050*
*Variation in laying date vs maximum temperature*				
Temperature	− 0.8 (0.9)	1, 34	− 0.8	0.42
Temperature^2^	− 0.1 (0.9)	1, 34	− 0.1	0.91
Temperature slope	− 1.5 (0.9)	1, 34	− 1.6	0.12
Temperature slope^2^	0.6 (0.9)	1, 34	0.6	0.52

### Sliding window analysis

Mean laying date was, in both species, best described by a model that included a negative quadratic response to mean maximum temperature (*p* = 0.057 and *p* = 0.050 respectively, Fig. 1b, Table 1, Table S1), in addition to the strong linear effect of mean maximum temperature (*p* < 0.0001 for both species, Fig. 1b, Table 1). For marsh tits, the window with highest support was from March 12th to April 26th and for blue tits from March 19^th^ to May 5^th^ (Table S1). While mean and maximum temperature were highly correlated across all time windows, the model with maximum temperature received the highest support (Table S1).

### Laying date distribution

The change in maximum temperature within the selected time windows was a significant predictor of laying date variation in blue tits, but not in marsh tits (temperature slope^2^ × species interaction: t = – 2.6, *p* = 0.012). In years with little change in maximum temperature (during the critical time window), blue tits started breeding over a larger part of the season (p < 0.0001, Table 1, Fig. 1c) but the variation in breeding start was not affected by temperature change within the selected time-window in marsh tits (*p* ≥ 0.12, Table 1, Fig. 1c). Overall maximum temperatures did not have an effect on the distribution of laying dates in either species (*p* ≥ 0.30, Table 1, Fig. 1d) and including the interaction between maximum temperature and its slope within the climate window did not improve the model fit for either blue tits (ΔAICc = 9.7, Table S2) or marsh tits (ΔAICc = 7.6, Table S2), i.e., the effect of change in maximum temperature on laying date variation in blue tits was not dependent on maximum temperature itself.

## Discussion

Blue and marsh tits advanced their laying date with 0.19 and 0.24 days per year during the study period, which is largely in line with previous estimates for this population (0.24 and 0.26, Källander et al. 2017). The relationship between maximum temperature and mean laying date was best described by a model that included a quadratic function. This implies that the ability to track future advancements in phenology with even warmer springs may start to erode, with potential future fitness declines due to increased mismatch between the food peak and nestling demand.

We did not find any effect of maximum spring temperature on variation in laying date, but egg laying in the blue tit population was more spread out in seasons when the temporal increase in spring temperature was slower. Marsh tits showed no such pattern. This could be a consequence of different selection pressures on breeding start linked to life-history variation in these species. Marsh tits form small flocks and defend territories in winter (Nilsson and Smith 1988). Establishment in a winter flock is dependent on being born early in the season (Nilsson and Smith 1988), because flocks quickly become saturated with defending, already established, conspecifics (Nilsson 1989, 1990). Thus, given that early hatching increases fitness of juvenile marsh tits, it is possible that there will still be selection for early laying in marsh tits in springs with stable temperature development. While blue tits also benefit from early laying in most years (Svensson 1997), early breeding in relation to conspecifics is likely not as important as it is for marsh tits.

Warmer spring temperatures can shorten the peak of food availability (Buse et al. 1999; Smith et al. 2011; Burger et al. 2012). In species with facultative second clutches, warmer springs have coincided with a decreased frequency of second clutches (Husby et al. 2009) and, hence, shorter breeding seasons (Hällfors et al. 2020; Møller et al. 2010). Thus, at the level of mean spring temperature, we predict that increasingly warmer springs under climate change will reduce the variation in breeding start. By contrast, our results show that in years with stable spring temperatures the breeding season can be prolonged, independent of mean temperature. Thus, temperature change in spring may be a previously overlooked key factor for variation in length of the breeding season. To determine if this is caused by changes in caterpillar phenology, future studies could benefit from investigating whether temperature change, and not only mean temperature, during spring affects the phenology and variation of the larval peak. Our study shows that different responses to changing spring temperatures can be observed even within sympatric and phylogenetically related species. However, as our data only cover two species, broader interspecific studies are needed to determine if life-history variation is a key driver of differences in the phenological response to temperature change. On a more general level this highlights the need for future analyses across species and life-history strategies when predicting the evolutionary responses to a warming climate.

## Supplementary Information

Below is the link to the electronic supplementary material.

Supplementary file1 (DOCX 1172 KB)

## Data Availability

Data and code are available in figshare: 10.6084/m9.figshare.19390769.v1.
