# Supplementary material for: Variation in breeding phenology in response to climate change in two passerine species
Source: Oecologia. 2022 Dec 22;201(1):279–85. doi: 10.1007/s00442-022-05306-5 (PMC9813050; doi:10.1007/s00442-022-05306-5)
Supplement: Supplementary file 1 — Supplementary file1 (DOCX 1172 KB) [file 442_2022_5306_MOESM1_ESM.docx]

**Electronic Supplementary Information to:**

**Variation in breeding phenology in response to climate change in two passerine species**

Fredrik Andreasson*, Andreas Nord, Jan-Åke Nilsson

Department of Biology, Section for Evolutionary Ecology, Lund University, Ecology Building, SE-223 62 Lund, Sweden

Corresponding and contact author*: fredrik.andreasson@biol.lu.se

**This supplement includes: Details on the *climwin* analyses, Table S1-2, Figures S1-4.**

***climwin***

Most studies using long-term datasets on breeding start rely on statistical tools to detect and analyze correlations between meteorological variables and phenology with the aim to identify the environmental cue/s that best explain first egg dates. Mean spring air temperature during a fixed time-window has commonly been used to evaluate the effect of warming on laying dates (e.g. Dunn and Winkler 1999, Visser et al 2003, Both et al 2004, Both and Visser 2005, Källander et al 2017). However, other characteristics such as minimum (Jankowiak 2014) or maximum temperature (Charmantier et al 2008), temperature change over a given period (Schaper et al 2012), or precipitation (Irons et al 2017) have also been found to significantly influence laying date. In addition, the time-window that best predicts laying date could be located outside of the fixed window often being used and can differ substantially between populations of the same species (Husby et al 2010). Therefore, we adopted a sliding-window approach, using the R package *climwin* (Bailey and van de Pol 2016, van de Pol et al 2016). Essentially, *climwin* allows for comparison between different environmental variables in all possible time-windows over the year to identify the time-window and aggregate statistics that best explains variation in phenology (laying date) based on AICc, Akaike Information Criterion (Akaike 1973) with small sample size correction.

We used daily mean, maximum and minimum air temperatures (°C), and daily precipitation (mm d^-1^), recorded by a weather station in Lund, approximately 20 km away from the study site around Lake Krankesjön in southernmost Sweden (55°42ʹN, 13°28ʹE) (Swedish Meteorological and Hydrological Institute, 2021, unpublished data). We then evaluated both mean values and slope (linear relationship between weather variables and date in the current window) of these weather variables and their linear and quadratic relationship with mean laying date. Model residuals were weighted by the inverse of the standard error of mean laying date to account for any uncertainty in this variable, primarily to account for yearly differences in sample size. We allowed time-windows to range from January 1^st^ to June 30^th^ to capture any potential early weather effects during winter and early spring. We did this separately for the two study species, blue tits (*Cyanistes caeruleus*) and marsh tits (*Poecile palustris*). All model combinations are presented in Table S1.

We used the function randwin, which retains all biological information but removes the climate signal, to determine if any selected candidate signals occurred merely by chance. A p-value (P_ΔAICc_, number of randomizations = 100) was calculated based on these randomizations. This provides the probability of a selected candidate signal occurring by chance alone (Bailey and van de Pol 2016). For more information, see model diagnostics in Fig. S4.

**References**

Akaike, H. 1973. Information theory and an extension of the maximum likelihood principle. Proceedings of the 2nd international symposium on information theory, 267–281. Akaemiai Kiado, Budapest.

Bailey, L. D. and van de Pol, M. 2016. climwin: an R toolbox for climate window analysis. PLoS ONE 11:e0167980.

Both, C. and Visser, M. E. 2005. The effect of climate change on the correlation between avian life-history traits. Global Change Biology 11:1606–1613.

Both, C., Artemyev, A. V., Blaauw, B., Cowie, R. J., Dekhuijzen, A. J., Eeva, T., Enemar, A., Gustafsson, L., Ivankina, E. V., Järvinen, A. et al 2004. Large-scale geographical variation confirms that climate change causes birds to lay earlier. Proceedings of the Royal Society B 271:1657–1662.

Charmantier, A., McCleery, R. H., Cole, L. R., Perrins, C., Kruuk, L. E. B. and Sheldon, B. C. 2008. Adaptive phenotypic plasticity in response to climate change in a wild bird population. Science 320:800–803.

Dunn, P. O. and Winkler, D. W. 1999. Climate change has affected the breeding date of tree swallows throughout North America. Proceedings of the Royal Society B 266:2487–2490.

Husby, A., Nussey, D. H., Visser, M. E., Wilson, A. J., Sheldon, B. C. and Kruuk, L. E. B. 2010. Contrasting patterns of phenotypic plasticity in reproductive traits in two great tit (*Parus major*) populations. Evolution 64:2221–2237.

Irons, R. D., Scurr, A. H., Rose, A. P., Hagelin, J. C., Blake, T. and Doak, D. F. 2017. Wind and rain are the primary climate factors driving changing phenology of an aerial insectivore. Proceedings of the Royal Society B 284: 20170412.

Jankowiak, L., Pietruszewska, H. and Wysocki, D. 2014. Weather conditions and breeding season length in blackbird (*Turdus merula*). Folia Zoologica 63:245–250.

Källander, H., Hasselquist, D., Hedenström, A., Nord, A., Smith, H. G. and Nilsson, J-Å. 2017. Variation in laying date in relation to spring temperature in three species of tits (Paridae) and pied flycatchers *Ficedula hypoleuca* in southernmost Sweden. Journal of Avian Biology 48:83–90.

Schaper, S. V., Dawson, J., Sharp, P. J., Gienapp, P., Caro, S. P. and Visser, M. E. 2012. Increasing temperature, not mean temperature, is a cue for avian timing of reproduction. American Naturalist 179:E55–E69.

van de Pol, M., Bailey, L. D., McLean, N., Rijsdijk, L., Lawson, C. R. and Brouwer, L. 2016. Identifying the best climatic predictors in ecology and evolution. Methods in Ecology and Evolution 7:1246–1257.

Visser, M. E., Adriaensen, F., van Balen, J. H., Blondel, J., Dhondt, A. A., van Dongen, S., du Feu, C., Ivankina, E. V., Kerimov, A. B., de Laet, J. et al 2003. Variable responses to large-scale climate change in European Parus populations. Proceedings of the Royal Society B 270:367–372.

**Table S1.** Output from the *climwin*-analyses. All models were based on mean laying date and both linear and quadratic response functions were included. Columns ‘Open’ and ‘Close’ indicate how many days before the reference day (30^th^ of June) the best time-window opened and closed, respectively. ΔAICc evaluates how much better the model performs compared to a null model (lower = better). Selected models with lowest ΔAICc are indicated in bold.

| Species | Climate signal | Summary  statistics | Response  function | ΔAICc | Open | Close |
| --- | --- | --- | --- | --- | --- | --- |
| **Blue tit** | **Daily maximum temperature** | **Mean** | **Quadratic** | **-63.01** | **103** | **56** |
| Blue tit | Daily maximum temperature | Mean | Linear | -61.63 | 104 | 56 |
| Blue tit | Daily mean temperature | Mean | Linear | -52.96 | 105 | 55 |
| Blue tit | Daily mean temperature | Mean | Quadratic | -51.04 | 102 | 55 |
| Blue tit | Daily minimum temperature | Mean | Linear | -20.06 | 107 | 63 |
| Blue tit | Daily maximum temperature | Slope | Quadratic | -19.68 | 139 | 113 |
| Blue tit | Daily minimum temperature | Mean | Quadratic | -19.59 | 110 | 65 |
| Blue tit | Daily minimum temperature | Slope | Quadratic | -15.07 | 144 | 99 |
| Blue tit | Daily mean temperature | Slope | Quadratic | -14.95 | 144 | 101 |
| Blue tit | Daily mean temperature | Slope | Linear | -12.11 | 79 | 45 |
| Blue tit | Daily precipitation | Slope | Quadratic | -11.67 | 170 | 155 |
| Blue tit | Daily maximum temperature | Slope | Linear | -11.30 | 71 | 46 |
| Blue tit | Daily precipitation | Slope | Linear | -9.75 | 143 | 55 |
| Blue tit | Daily minimum temperature | Slope | Linear | -9.75 | 108 | 87 |
| Blue tit | Daily precipitation | Mean | Quadratic | -8.80 | 68 | 68 |
| Blue tit | Daily precipitation | Mean | Linear | -8.22 | 99 | 55 |
| **Marsh tit** | **Daily maximum temperature** | **Mean** | **Quadratic** | **-69.83** | **110** | **65** |
| Marsh tit | Daily maximum temperature | Mean | Linear | -69.01 | 110 | 68 |
| Marsh tit | Daily mean temperature | Mean | Linear | -64.24 | 107 | 67 |
| Marsh tit | Daily mean temperature | Mean | Quadratic | -62.04 | 107 | 67 |
| Marsh tit | Daily minimum temperature | Mean | Linear | -23.40 | 107 | 67 |
| Marsh tit | Daily minimum temperature | Mean | Quadratic | -21.17 | 110 | 68 |
| Marsh tit | Daily mean temperature | Slope | Quadratic | -18.58 | 144 | 100 |
| Marsh tit | Daily minimum temperature | Slope | Quadratic | -18.21 | 146 | 99 |
| Marsh tit | Daily precipitation | Mean | Quadratic | -16.46 | 170 | 162 |
| Marsh tit | Daily maximum temperature | Slope | Quadratic | -15.94 | 108 | 42 |
| Marsh tit | Daily precipitation | Slope | Quadratic | -14.19 | 170 | 157 |
| Marsh tit | Daily mean temperature | Slope | Linear | -13.38 | 81 | 45 |
| Marsh tit | Daily maximum temperature | Slope | Linear | -12.20 | 109 | 42 |
| Marsh tit | Daily precipitation | Mean | Linear | -12.08 | 170 | 169 |
| Marsh tit | Daily precipitation | Slope | Linear | -11.79 | 170 | 157 |
| Marsh tit | Daily minimum temperature | Slope | Linear | -11.49 | 108 | 45 |

**Table S2.** Comparisons between overall and species-specific models on variation in laying date (laying date variation) that include the interaction between mean maximum temperature within the selected climate window (temp) and the slope of change in daily maximum temperature during the same time window (slope) and models without the interaction. Models in bold were selected based on AICc and used as final models.

| **Species** | **Model** | **AICc** | **AIC** |
| --- | --- | --- | --- |
| Overall | Laying date variation ~ species * temp^2^ * slope^2^ | 217.51 | 204.40 |
|  | **Laying date variation ~ species * temp^2^ + species * slope^2^** | **202.14** | **198.14** |
|  |  |  |  |
| Blue tit | Laying date variation ~ temp^2^ * slope^2^ | 99.19 | 91.34 |
|  | **Laying date variation ~ temp^2^ + slope^2^** | **89.50** | **86.88** |
|  |  |  |  |
| Marsh tit | Laying date variation ~ temp^2^ * slope^2^ | 120.14 | 112.29 |
|  | **Laying date variation ~ temp^2^ + slope^2^** | **112.54** | **109.91** |

**Figure S1.** There was no correlation between mean maximum temperature within the identified climate window and the temporal increase in maximum temperature within the same time-window (i.e. temperature slope) in either blue tits (Pearson's r = 0.04, p = 0.79) or marsh tits (Pearson's r = -0.16, p = 0.34).

**
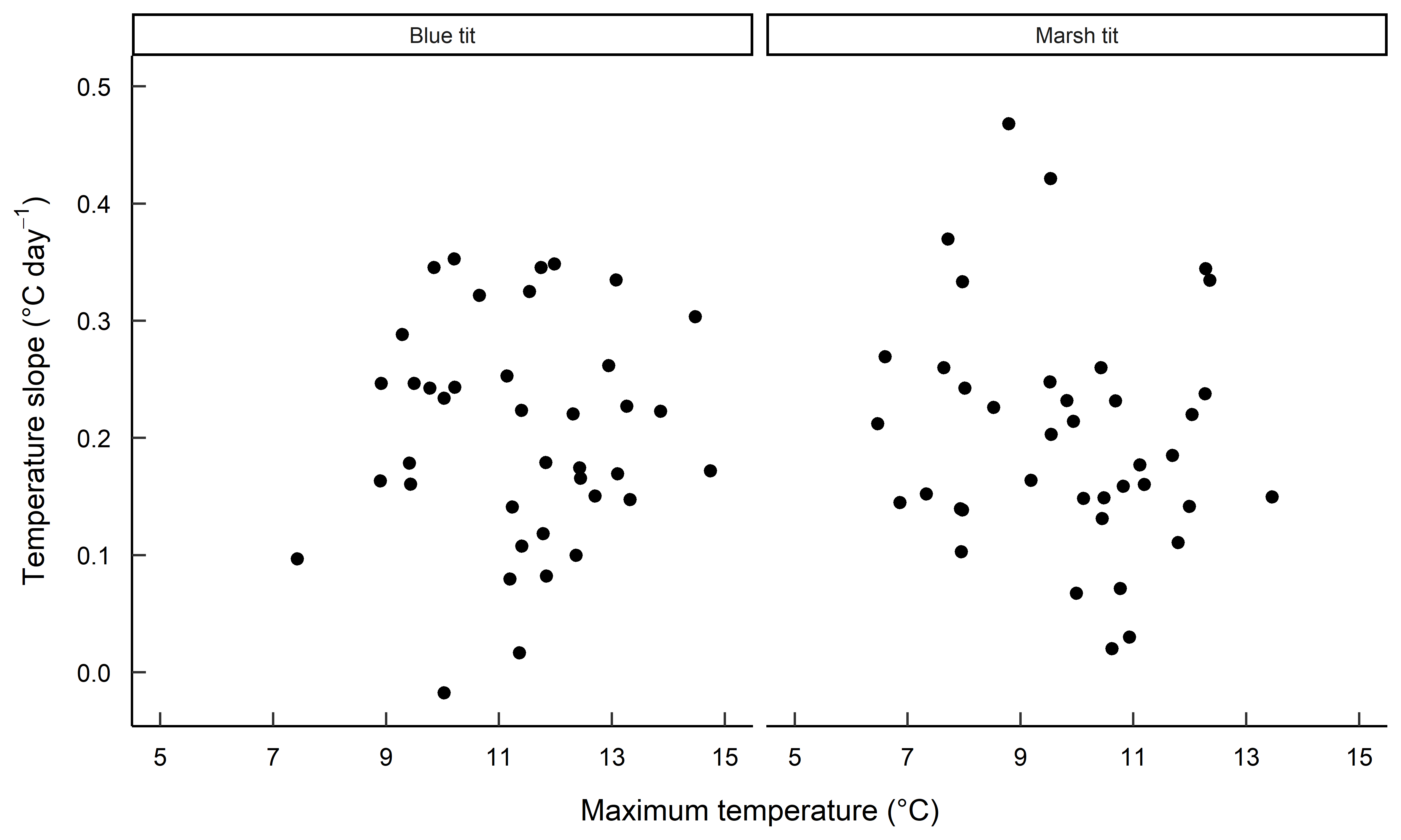
**

**Figure S2.** Distribution of laying dates (Laying date; 1 = April 1^st^) in blue tits (a) and marsh tits (b). Note the different scales on the y-axes between species.

**
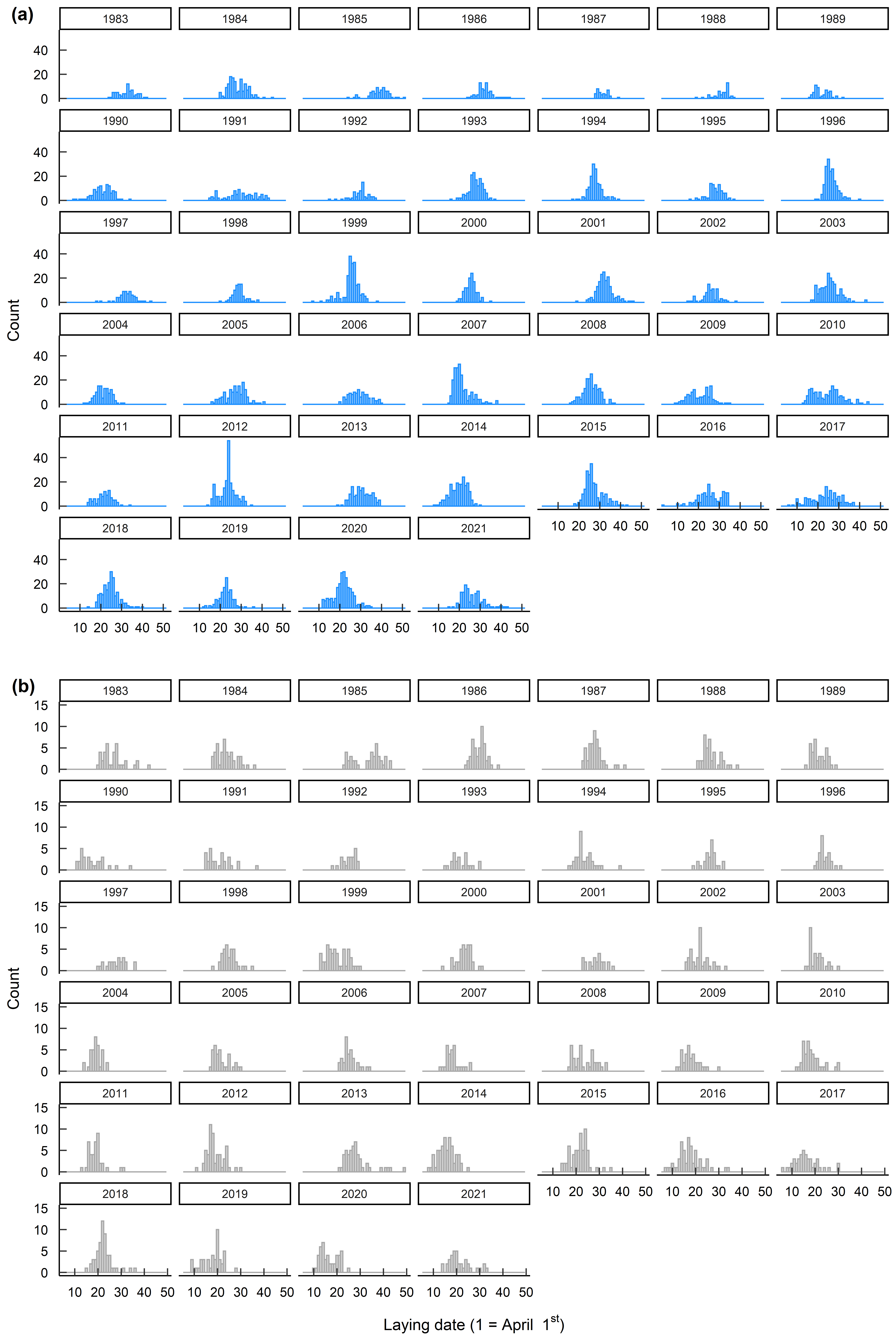
**

**Figure S3.** Maximum daily temperature (°C) in the selected climate window in blue tits (blue) and marsh tits (grey) across all years. Linear regressions for both species in all years are plotted in blue (blue tits) and black (marsh tits).


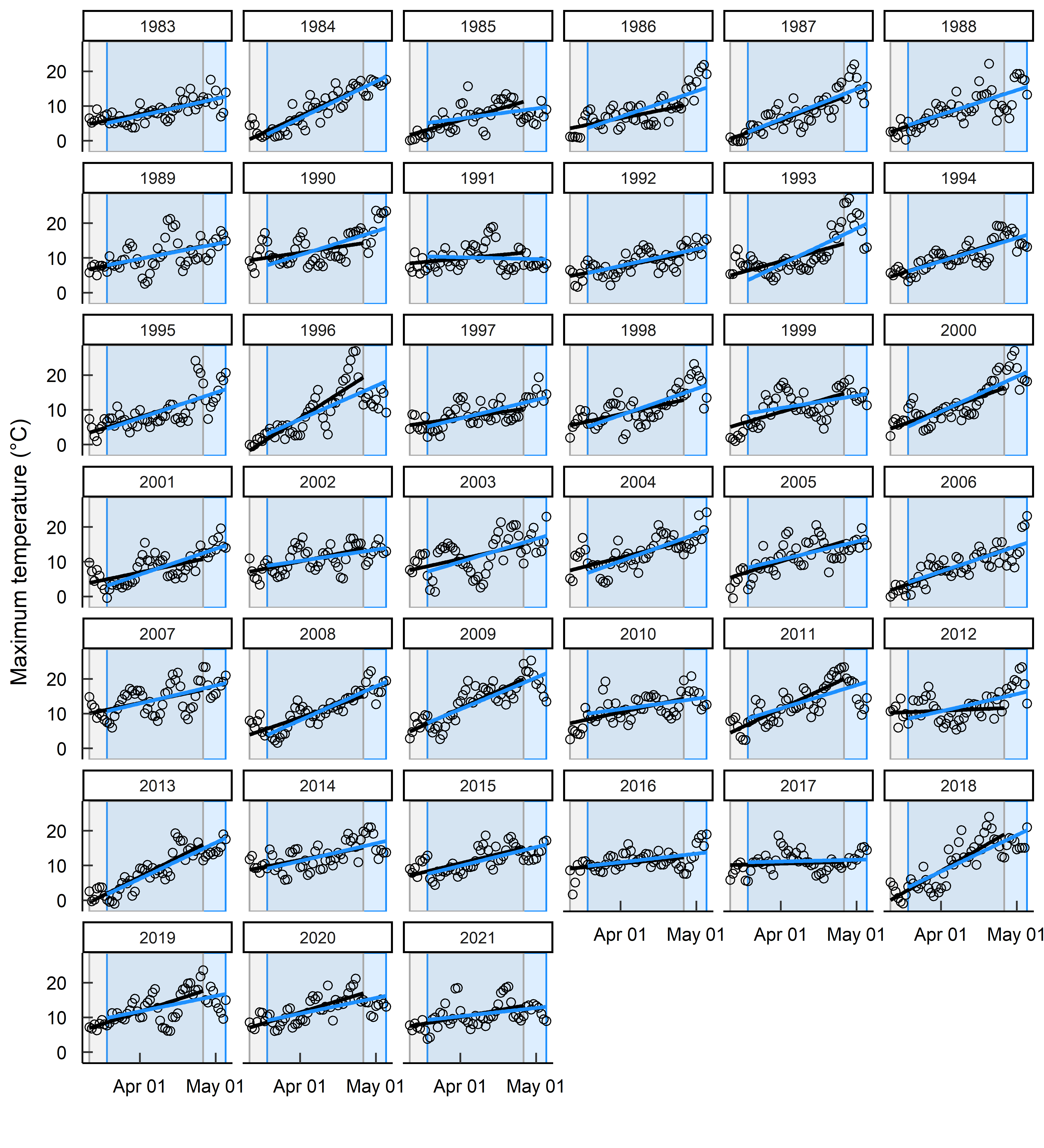


**Figure S4.** Model diagnostics for the *climwin*-analyses for blue tits (a) and marsh tits (b). From the top left, the panels show:

1. ΔAICc for the best environmental variable (maximum temperature) compared to the null model across all possible windows. Both species show only one “area” of optimal windows (red).
2. 95% confidence set of models, where we can be 95% certain that the “best” model falls within the shaded area.
3. Beta linear and beta quadratic shows the β-coefficient for the model across all possible windows (i.e. the coefficients for the best window are those presented in Table 1). Windows adjacent to the best model show similar coefficients.

From the bottom left:

1. ΔAICc of randomized data (histogram) compared to the obtained ΔAICc for the best supported model (dashed line). P_ΔAICc_ < 0.001 for both species indicates that it is highly unlikely that the obtained window was selected by chance.
2. 95% model confidence set (i.e., not just the best window, but including the 95% best models).
3. The resulting relationship between the environmental variable (maximum temperature) and biological response (mean laying date). Essentially, Fig. 1b in the main manuscript.

**Figure S4a.**

**
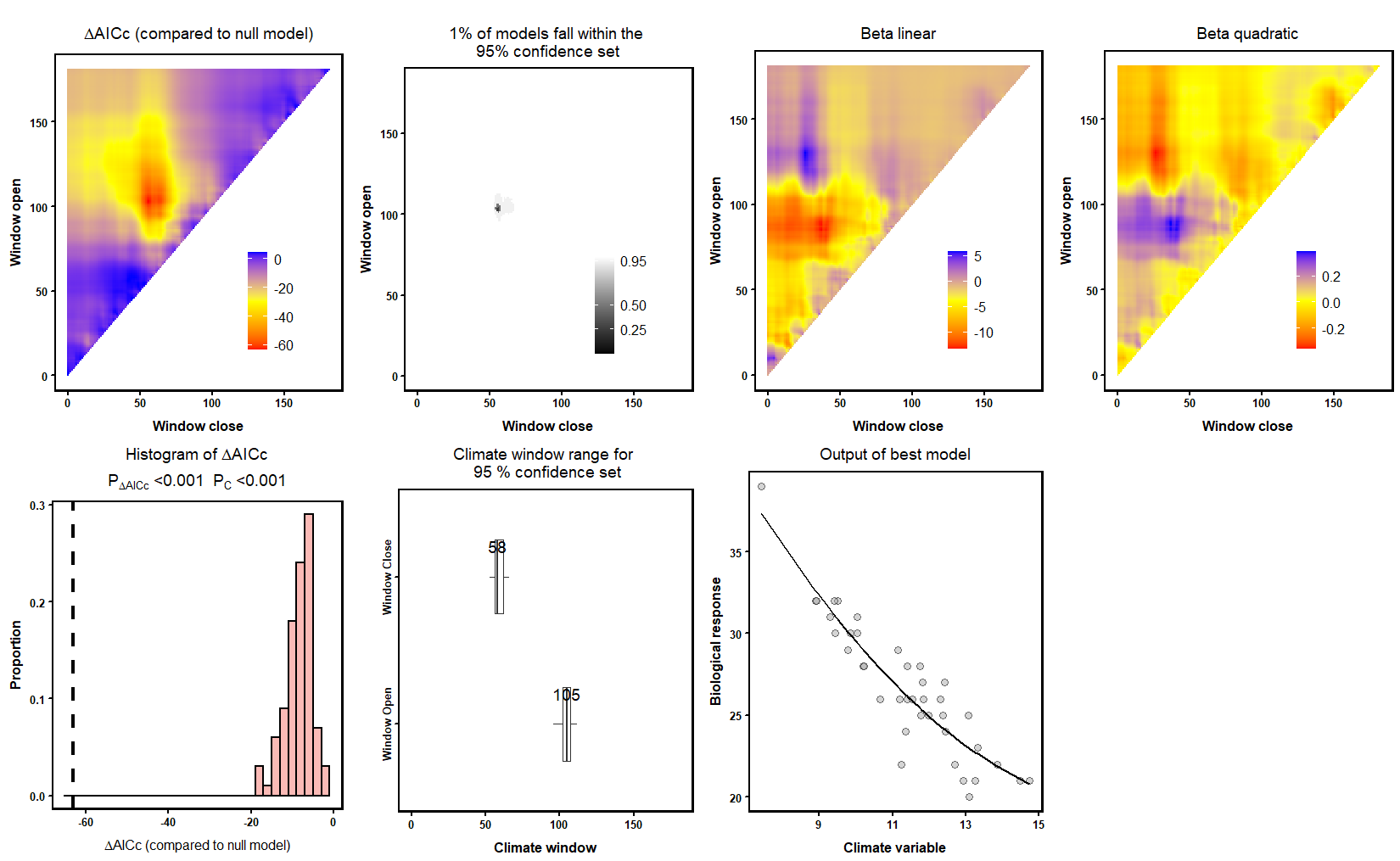
**

**Figure S4b.**

**
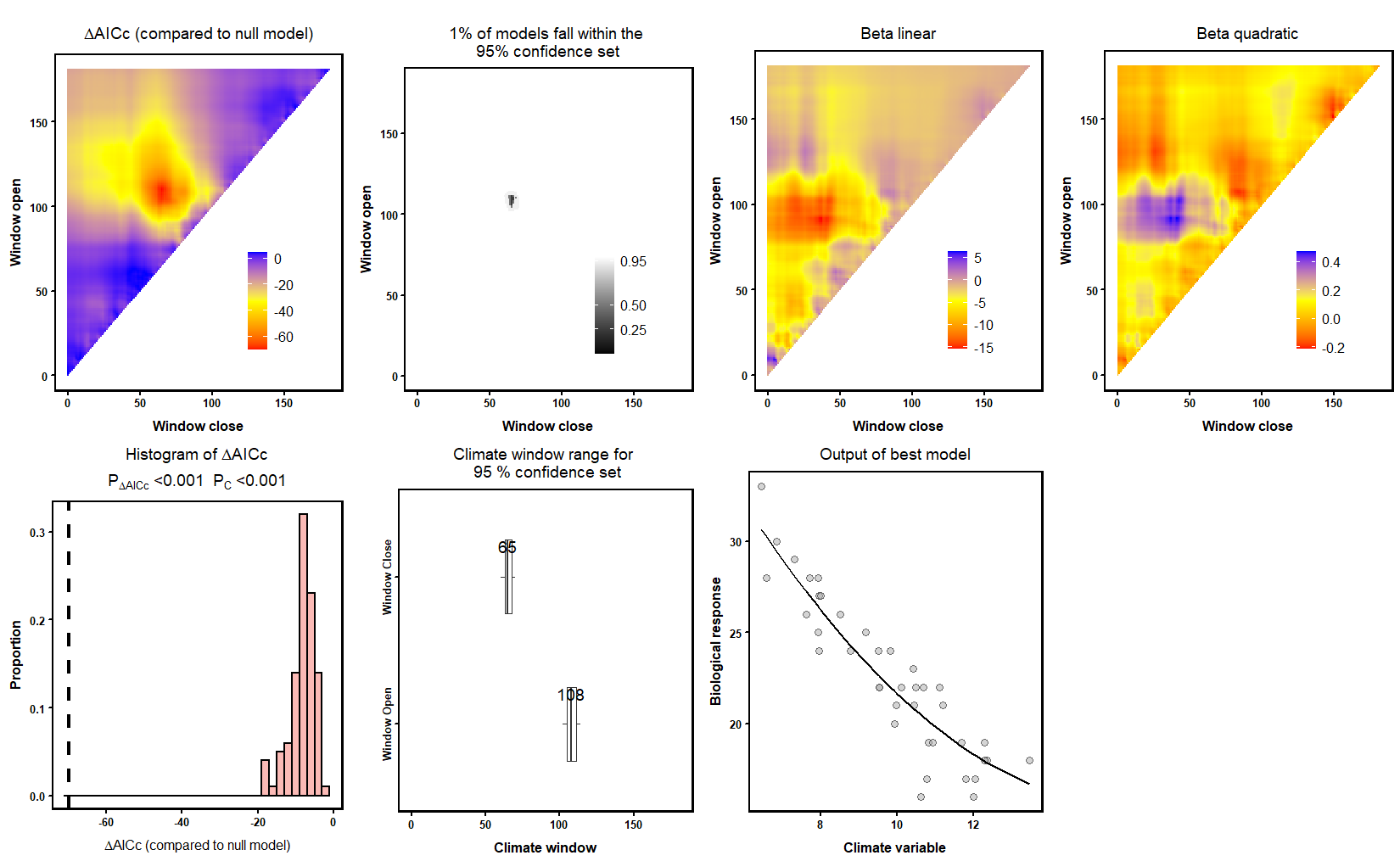
**
